# What Is the Mechanism Underlying the Interleaving Effect in Category Induction: An Eye-Tracking and Behavioral Study

**DOI:** 10.3389/fpsyg.2021.770885

**Published:** 2021-12-16

**Authors:** Yabo Ge, Fengying Li, Xinyu Li, Weijian Li

**Affiliations:** ^1^Department of Psychology, Zhejiang Normal University, Jinhua, China; ^2^College of Teacher Education, Zhejiang Normal University, Jinhua, China; ^3^Key Laboratory of Intelligent Education Technology and Application of Zhejiang Province, Zhejiang Normal University, Jinhua, China; ^4^Collaborative Innovation Center, Zhejiang Normal University, Jinhua, China

**Keywords:** attention attenuation hypothesis, discriminative-contrast hypothesis, category induction, interleaving effect, eye-tracking

## Abstract

Interleaved practice (i.e., exemplars from different categories are intermixed within blocks) has been shown to enhance induction performance compared to blocked practice (i.e., exemplars from the same category are presented sequentially). The main aim of the present study was to examine explanations of why interleaved practice produces this benefit in category induction (known as the interleaving effect). We also evaluated two hypotheses, the attention attenuation hypothesis and the discriminative-contrast hypothesis, by collecting data on participants’ fixation on exemplars, provided by eye-tracking data, and manipulating the degree of discriminative-contrast. In Experiments 1 and 2, participants were instructed to learn the style of 12 new artists in blocked and interleaved practice in fixed-paced and self-paced learning conditions, respectively. We examined fixation durations for six positions (temporal sequence of exemplars presented in each block) using eye-tracking. The results of the two experiments, based on eye-tracking data, suggested that attention attenuation may not be the primary mechanism underlying the interleaving effect in category induction. In Experiment 3, we manipulated the degree of discriminative-contrast to examine the impact on the interleaving effect in category induction. The results showed that the main effect of the degree of discriminative-contrast was significant, and performance in the high-contrast condition was significantly better than those in the medium-contrast and low-contrast conditions. Thus, the current results support the discriminative-contrast hypothesis rather than the attention attenuation hypothesis.

## Introduction

Learning through category induction is a primary method by which humans acquire knowledge (Kruschke, 2005; Brunmair and Richter, 2019). Category learning, also known as concept attainment, span from infancy through to adulthood, and include learning the label of novel objects (Vlach et al., 2008), different species of birds (Wahlheim et al., 2011), landscapes or skyscapes painted by different artists (Kornell et al., 2010), or different types of mathematics assignments (Taylor and Rohrer, 2010; Rohrer et al., 2015). To engage in category induction, individuals must be able to identify recurring patterns (Ashby and O'Brien, 2005; Eglington and Kang, 2017; Sana et al., 2017). For example, to identify a specific painter’s style, people must be able to identify patterns that differ from those present in paintings by others (Kornell and Bjork, 2008; Kang and Pashler, 2012; Guzman-Munoz, 2016). Since this form of learning is critical to individuals making sense of their environment, numerous studies have focused on how to improve category induction.

A convincing body of evidence demonstrates that the sequence of exemplar presentations (i.e., interleaved or blocked practice) has a significant impact on category induction performance (Kornell and Bjork, 2008; Vlach et al., 2008; Kornell et al., 2010; Wahlheim et al., 2011; Kang and Pashler, 2012; Zulkiply et al., 2012; Birnbaum et al., 2013; Zulkiply and Burt, 2013; Carvalho and Goldstone, 2014; Rohrer et al., 2015; Mathy and Feldman, 2016; Eglington and Kang, 2017; Foster et al., 2019). When stimuli are presented in blocked practice, exemplars of the same category are presented sequentially. In contrast, in interleaved practice, exemplars from different categories are intermixed within blocks. Thus, exemplars of the same category are not presented until after many exemplars from other categories have been presented. Many studies have suggested that there are benefits to interleaved practice in category learning (Kornell and Bjork, 2008; Vlach et al., 2008; Kornell et al., 2010; Wahlheim et al., 2011; Kang and Pashler, 2012; Rohrer, 2012; Dunlosky et al., 2013; Verkoeijen and Bouwmeester, 2014; Rohrer et al., 2015; Metcalfe and Xu, 2016; Brunmair and Richter, 2019), which is referred to as the interleaving effect (Zulkiply and Burt, 2013). Kornell and Bjork (2008) found that interleaved practice was superior to blocked practice in category induction tasks involving young adults’ memory of 12 artists’ styles. In this study, each artist was represented by 10 paintings, which were landscapes, and participants learned six of them, and the remaining four paintings were used in the testing phase. During the learning phase, the blocked practice was achieved by the paintings of six artists being presented in individual blocks, in which each of the six paintings by the same artist was presented in a single block. For interleaved practice, the paintings of six different artists were presented in blocks consisting of six paintings from six different artists. The blocked and interleaved practice conditions were manipulated within participants. The paintings, on which the artists’ names were displayed, were presented for 3 s. During the testing phase, participants were instructed to classify a new range of paintings by the original artist. The results indicated that participants’ classifications of the new paintings were more accurate for artists for whom the paintings had been presented in interleaved practice than in blocked practice. The interleaving effect has been replicated many times (Kornell et al., 2010; Wahlheim et al., 2011; Kang and Pashler, 2012; Birnbaum et al., 2013; Verkoeijen and Bouwmeester, 2014; Metcalfe and Xu, 2016). For example, Birnbaum et al. (2013) used pictures of butterflies from different species to evaluate the acquisition of categories representing natural species using interleaved and blocked practice, and also confirmed the presence of the interleaving effect. Kornell et al. (2010) reported comparable results for older adults, and Wahlheim et al. (2011) demonstrated similar findings using pictures of diverse types of birds, rather than landscapes. Despite growing evidence that interleaving is beneficial, the mechanisms underlying category induction are not yet fully understood.

One influential explanation underlying the interleaving effect, the attention attenuation hypothesis, postulates that blocked practice may impair learning by reducing the amount of attention participants pay to each of the repeated presentations. This is because exemplars in a block are from the same category and are therefore highly similar (Hintzman, 1974). Successive presentation of highly similar exemplars could create a high level of familiarity, or fluency. Consequently, participants may pay less attention to the exemplars because they subconsciously overestimate the extent to which they have mastered the category (Zechmeister and Shaughnessy, 1980). Attention is also likely to be diminished when sequentially repeating similar exemplars in blocks, as this may lead to habituation. To date, a small number of studies have investigated the attention attenuation hypothesis with category learning, and the results have been equivocal. Kornell et al. (2010) examined the interleaving effect using two learning tasks: an artist’s paintings displayed six times, either in blocked or interleaved practice, divided into two tasks: (a) the induction task in which participants studied the various paintings without any painting being repeatedly displayed, and then underwent a test; and (b) the repetition task in which participants studied a single painting by each artist, and then underwent a test. The attention attenuation hypothesis suggests that blocked practice may impair learning by reducing the amount of attention individuals allocate to continuous presentations since the exemplars are highly similar (Hintzman, 1974). The exemplar’s similarity in the repetition task of Kornell et al. (2010) was far greater than that for the induction task. Thus, the researchers predicted that there was greater attention attenuation in the repetition task than in the induction task, which should result in a larger interleaving effect in the repetition task (Kornell et al., 2010). In fact, the magnitude of the interleaving effect did not differ significantly between the repetition and induction tasks, and the results did not support the attention-attenuation hypothesis.

Conversely, Wahlheim et al. (2011) found supporting evidence for the attention-attenuation hypothesis. These researchers studied memory performance at a test point for items that had been previously studied. Memory performance was identified as the indicator of when attention had been paid to the position of the item in the study sequence. The results demonstrated that memory performance for exemplars in each position within an individual block decreases as a function of relative position in blocked practice, whereas no such decrease would arise for interleaved practice blocks. A possible explanation was that, in later presentations of the blocked sequences, participants did not attend to the items to the same extent as they did the earlier items; therefore, the items were not recalled as readily. We reasoned that the amount of attention to items studied cannot be fully measured by memory performance at the time of testing, which is an indirect indicator of attention. By measuring either the timing of gaze or the motion of the eyes relative to the head. In such cases, eye-tracking systems are suitable for cognitive and behavioral experiments, including this type of investigation into category induction (Kruschke et al., 2005; Rehder and Hoffman, 2005a,b; Carvalho and Goldstone, 2017; Zaki and Salmi, 2019).

Fixation time, measured by eye-tracking, is a common attention allocation index in cognitive psychology experiments. For example, Carvalho and Goldstone (2017) used eye-tracking to support that interleaved practice highlights the variations between presented categories, whereas blocked practice highlights the commonalities within one category. We adopted a fixation duration measurement using an eye-tracking system as a more direct indicator of exemplar study duration. As mentioned, the current investigation (Experiments 1 and 2) explores the attention attenuation hypothesis in more depth by using fixation duration times collected by eye-tracking equipment as an indicator of practice time. The attention attenuation hypothesis suggests that the fixation durations for exemplars are expected to be greatest for the first exemplars in the blocked practice blocks, and to decline for subsequent exemplars. Little or no change is expected for the interleaved practice blocks.

An alternative to the attention attenuation hypothesis concerning the interleaving effect is the discriminative-contrast hypothesis which can be applied from the perspective of interleaved practice (Kornell and Bjork, 2008; Kang and Pashler, 2012; Birnbaum et al., 2013; Eglington and Kang, 2017). In the light of the discriminative-contrast hypothesis, observing the differences between categories is crucial to category induction. Thus, interleaved practice results in exemplars of different categories being intermixed within blocks, which is conducive to learning the differences between them. Consequently, the performance of category induction for interleaved practice was superior to that for blocked practice. Specifically, alternately presenting examples from different categories could highlight the differences between categories through comparison. Thus, under these circumstances, participants would be able to learn categories more readily and improve classification performance (Wahlheim et al., 2011; Kang and Pashler, 2012; Birnbaum et al., 2013). This hypothesis is consistent with that of Yamauchi and Markman (2000) and Markman and Ross (2003), who demonstrated that classification learning is more sensitive to differences between categories.

Kang and Pashler (2012) addressed the question of whether the advantages of interleaved practice as a result of increased temporal spacing between the paintings presented which were by the same artist or as a result of the interleaving of paintings by the different artists. The researchers manipulated three experimental conditions: massed, interleaved, and simultaneously different (a screen displaying two different types of paintings to make the differences between the categories more salient). The simultaneously different conditions yielded significantly better learning outcomes than the blocked and interleaved conditions did. In addition, the category induction was virtually unaffected by temporal spacing. However, Foster et al. (2019) examined both the discriminative-contrast hypothesis and the distributed-practice through testing participants’ performance on the calculation of mathematical problems, namely, the volume of three-dimensional geometric shapes. Specifically, four learning settings were used in the study: standard blocked, standard interleaved, remoted blocked, and remote interleaved. The results indicated that interleaved practice can benefit the learning of mathematical materials and that participants benefited nearly equally from interleaving whether it presented in the standard interleaved practice or the remote interleaved practice conditions. These findings suggest that interleaving effects involved in geometry problems may be largely driven by distributed practice rather than explicable by the discriminative-contrast hypothesis. However, based on current research, category learning of paintings’ styles, which is more likely to represent a visual task, may be different from mathematical tasks. For visual materials, the discriminative-contrast effect may be essential to the interleaving effect in category induction. To test our hypothesis that paintings learning is sensitive to discriminative-contrast effects, Experiment 3 attempted to directly manipulate the degree of discriminative-contrast in three levels: low-contrast, medium-contrast, and high-contrast conditions. The purpose is to evaluate to what extent the degree of discriminative-contrast affects classification performance in category induction tasks and to provide more direct, convergent evidence for the discriminative-contrast hypothesis. This study predicts that if the discriminative contrast is beneficial to the interleaving effect, then the degree of discriminative-contrast presence affects the classification performance.

## Experiment 1

Experiment 1 was designed to provide eye-tracking to test the testing mechanism for the attention attenuation hypothesis. Per the attention attenuation hypothesis, the blocked practice may impair learning by reducing the amount and salience of attention the participant gives to repeated presentations. This is because exemplars in a block are from the same category and are, therefore, highly similar. Therefore, testing for a significant difference in fixation durations at six positions in blocked and interleaved practice blocks using category learning tasks can help further understanding of this hypothesis. Participants in this experiment were asked to view 72 paintings by 12 artists and learn the different styles. Paintings by six of the artists were presented as blocked practice (presenting different paintings of the same artist in each block), and paintings by the remaining six artists were presented as interleaved practice (mixing paintings by different artists in each block).

### Method

#### Participants and Design

The sample size was determined by a power analysis based on predicted effect size, using G*power 3.0 (Faul et al., 2007, 2009). Based on the predicted effect size (*η*^2^ = 0.20) and the targeted power (*β* = 0.80), the power analysis suggested that a sample size of 28 was required. Twenty-nine participants (22 women and seven men) were recruited from Zhejiang Normal University and received course credit and a gift in return for their participation. Participants’ mean age was 21.93 years (*SD* = 1.85), and all had a normal or corrected-to-normal vision.

The research procedure for this experiment and subsequent experiments conformed to the ethical standards of the 1964 Declaration of Helsinki. The Zhejiang Normal University Review Board approved the research procedures and an informed consent form was signed by each participant before the experiment.

#### Materials and Apparatus

**Figure 1:**
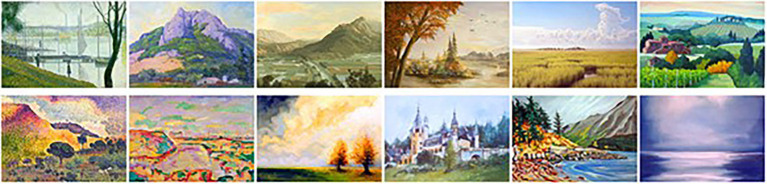
Landscapes and skyscapes used in Experiment 1, reprinted from “Learning Concepts and Categories: Is Spacing the ‘Enemy of Induction?’” by Kornell and Bjork (2008). Copyright 2008 by Kornell. Reprinted with permission.

The materials included 120 landscapes or skyscapes painted by 12 artists (i.e., 10 paintings by each of the following artists: Georges Braque, Henri-Edmond Cross, Judy Hawkins, Philip Juras, Ryan Lewis, Marilyn Mylrea, Bruno Pessani, Ron Schlorff, Georges Seurat, Ciprian Stratulat, George Wexler, and Yie Mei). The paintings were taken from the similar study of Kornell and Bjork (2008) (Figure 1). These artists were selected because they were not well-known. The artists’ names were translated into Chinese for the convenience of Chinese participants. Six paintings by each artist were presented during the study phase and four were presented during the test phase. All of the painting files were in .jpg format (500 × 350 pixels) and resized to produce a 17.86 cm × 12.50 cm rectangle on the computer screen. Participants’ eye movements were recorded in the study phase using a Tobii 1750 eye-tracking system sampling at 50 Hz and category induction performance was recorded in E-prime 2.0.

#### Procedure

This experiment was conducted using E-prime 2.0 and Tobii Studio eye-tracking software. Participants completed two separate phases: the study phase and the test phase. During the study phase, participants learned the different styles of the 12 artists by studying six of each artist’s paintings. The 12 artists were randomly divided into two groups of six and appeared equally frequently in the blocked and interleaved practice conditions. In blocked practice (B) condition, six paintings by a single artist were presented in each block. In the interleaved practice (I) condition, one painting by each of the six artists was presented in each block. Each painting appeared on the computer screen for 3 s, and the artist’s name was displayed below the painting. The order of the blocks was as follows: B-I-I-B-B-I-I-B-B-I-I-B (as per Kornell and Bjork, 2008). During the test phase, 48 test trials were divided into four blocks of 12 paintings each. Each block consisted of 12 new paintings, with one representing each of the 12 artists, in random order. In the test trials, an unfamiliar painting by one of the 12 artists was presented, along with all the artists’ names below the paintings. Participants signified the name of the artist whom they believed had created each painting by pressing the corresponding button using a keyboard.

### Results

#### Category Induction Performance

**Figure 2:**
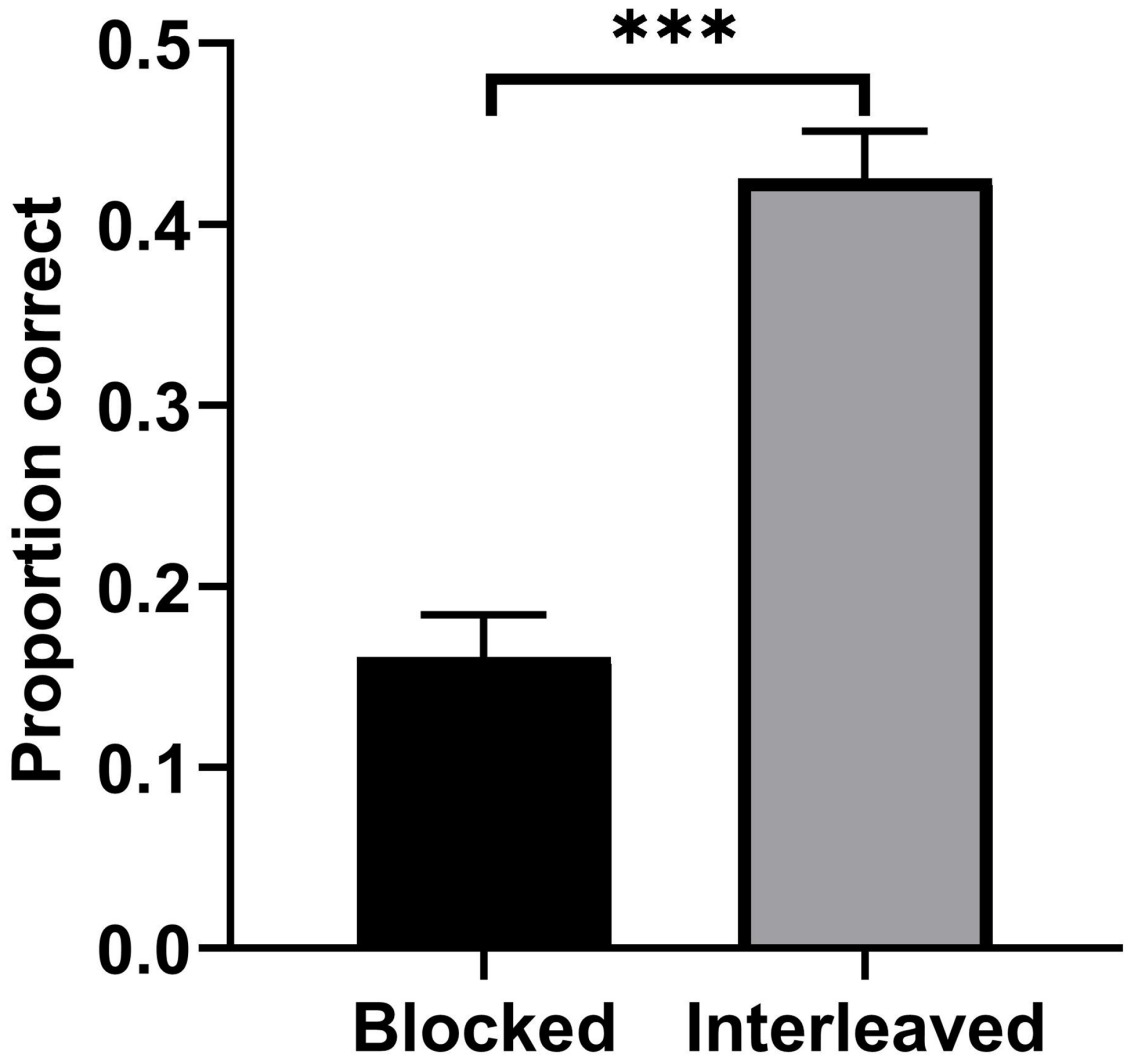
Mean test performance as a function of study condition in Experiment 1. Error bars represent SEM. ^***^p < 0.001.

Figure 2 displays the mean performance on the final category test as a function of study condition. Category induction performance was compared between the two conditions using a paired-samples *t*-test. Participants’ performance for interleaved practice (*M* = 0.43, *SD* = 0.14, 95% CI [0.37, 0.48]) was significantly superior to that observed in blocked practice (*M* = 0.16, *SD* = 0.13, 95% CI [0.11, 0.21]), *t*(28) = 9.08, *p* < 0.001, Cohen’s *d* = 1.46. This result indicated that the interleaving effect was present in category induction.

#### Fixation Duration

**Figure 3:**
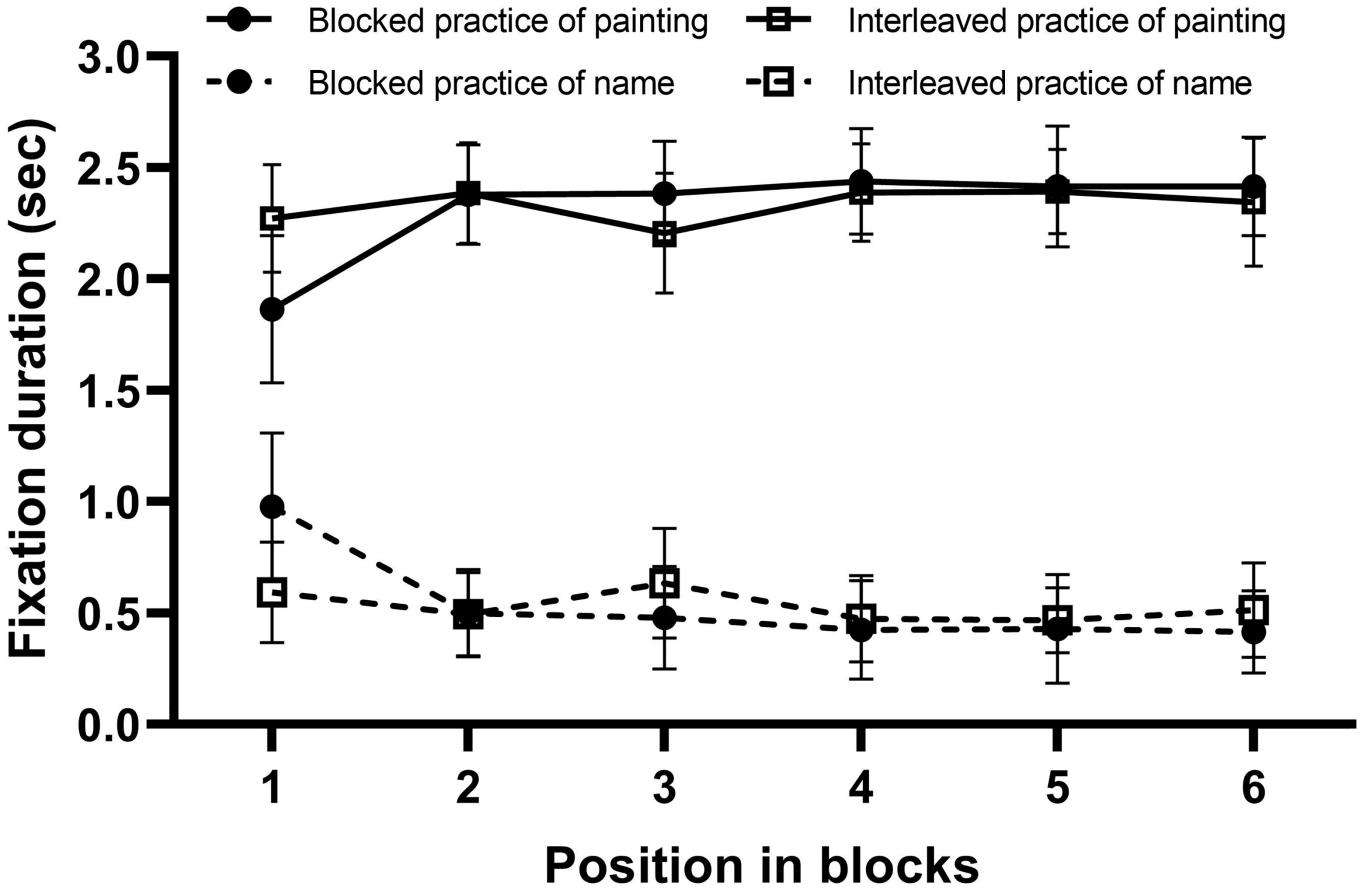
Mean fixation durations for the paintings as a function of relative position and study condition in Experiment 1. Error bars represent SEM.

The researchers focused mainly on the two interest areas, namely, the stimulating area contained the paintings and the artists’ names, respectively. Next, the fixation duration within the interest areas of the paintings and names was calculated. Figure 3 shows the mean fixation durations for the paintings’ and names’ interest areas as a function of relative position and study condition. A 2 (stimulus: painting, name) × 2 (study condition: blocked, interleaved) × 6 (exemplar position: 1, 2, 3, 4, 5, and 6) ANOVA was performed.

The main effect of stimulus was significant, *F*(1, 28) = 712.50, *p* < 0.01, *η_p_*^2^ = 0.96. Fixation duration in paintings (*M* = 2.32, *SD* = 0.20) was significantly superior to fixation duration in names (*M* = 0.53, *SD* = 0.17). Critically, the main effect of the study condition was not statistically significant, *F*(1, 28) = 0.66, *p* = 0.42, *η_p_*^2^ = 0.023, indicating that fixation durations were not significantly different between blocked and interleaved practice. Furthermore, to test for evidence for the null effect of the study condition, we computed a Jeffreys–Zellner–Siow (JZS) Bayes factor using the ANOVA function in JASP (https://jasp-stats.org/; Wagenmakers et al., 2010; Dienes, 2014; Marsman and Wagenmakers, 2017; Hu Chuan-Peng et al., 2018). The Bayes factor was BF_01_ = 11.73, indicating that the observed data are 11.73 times more likely under the null hypothesis (which postulates an absence of an effect) than under the alternative hypothesis (which postulates the presence of an effect). Thus, the data provided substantial evidence in favor of a null effect of the study condition (Jarosz and Wiley, 2014). Moreover, the main effect of exemplar position was also not statistically significant, *F*(5, 140) = 1.88, *p* = 0.13, *η_p_*^2^ = 0.063.

The interaction between stimulus and study condition was also not significant, *F*(1, 28) = 0.34, *p* = 0.57, *η_p_*^2^ = 0.12. The interaction between study condition and exemplar position was not significant, *F*(5, 140) = 0.99, *p* = 0.42, *η_p_*^2^ = 0.034. In addition, the interaction between stimulus and exemplar position was significant, *F*(5, 140) = 44.53, *p* < 0.01, *η*^2^ = 0.61. Simple effects analysis showed that fixation durations for the six relative positions differed significantly in painting, *F*(5, 24) = 41.23, *p* < 0.001, *η*^2^ = 0.60, Position 1 was significantly shorter relative to those observed for Positions 2, 3, 4, 5, and 6, and the fixation duration for Position 2 was significantly longer relative to those observed for Positions 1 and 3; fixation durations for the six relative positions differed significantly in name, *F*(5, 24) = 43.61, *p* < 0.001, *η*^2^ = 0.61, however, Position 1 was significantly longer relative to those observed for Positions 2, 3, 4, 5, and 6, and the fixation duration for Position 2 was significantly shorter relative to those observed for Positions 1, 3, and 6. The interaction between stimulus, study condition, and exemplar position were significant, *F*(5, 140) = 30.21, *p* < 0.01, *η*^2^ = 0.52. Simple and simple effects analysis showed that fixation durations for the six relative positions differed significantly in blocked study of painting, *F*(5, 24) = 76.79, *p* < 0.001, *η*^2^ = 0.73, Position 1 was significantly shorter relative to those observed for Positions 2, 3, 4, 5, and 6; fixation duration for the six relative positions differed significantly in blocked study of name, *F*(5, 140) = 71.58, *p* < 0.001, *η*^2^ = 0.72, Position 1 was significantly longer relative to those observed for Positions 2, 3, 4, 5, and 6; fixation durations for the six relative positions differed significantly in interleaved study of painting, *F*(5, 140) = 6.18, *p* < 0.001, *η*^2^ = 0.18, Position 1 was significantly shorter relative to those observed for Positions 2, 4, and 5; fixation durations for the six relative positions differed significantly in interleaved study of name, *F*(5, 140) = 6.40, *p* < 0.001, *η*^2^ = 0.19, Position 1 was significantly longer relative to those observed for Positions 2, 4, and 5. The results show that there was no downwards tendency of stimulus’ fixation duration across positions in the blocked study, the attention attenuation hypothesis was not supported. However, there was an ascending tendency in the blocked study of painting and a downwards tendency in the blocked study of a name, so we cannot rule out the influence of a name’s study on the interleaved effect.

### Discussion

This experiment tested the attention attenuation hypothesis, which was not supported by the eye-tracking data. However, the experiment involved fixed-paced learning, where stimuli were presented for only 3 s, and coding may have been insufficient for some stimuli. Therefore, the participants might not have had the opportunity to reduce their attention. To compensate for this, we examined the attention attenuation hypothesis using self-paced learning to produce convergent evidence. If the fixation durations for the six relative positions showed a decreasing trend in the blocked study, but not in the interleaved study, and fixation durations in the interleaved study were longer relative to those observed in the blocked study, the findings would support this hypothesis.

## Experiment 2

### Method

#### Participants and Design

Twenty-five participants (20 women, five men) were recruited from Zhejiang Normal University and received course credit and a gift for their participation. Participants’ mean age was 19.08 years (*SD* = 1.68), and they all had a normal or corrected-to-normal vision. The design of this experiment was the same as that of Experiment 1, which was a within-subjects design.

#### Materials, Apparatus, and Procedure

The materials, apparatus, and procedure used in this experiment were the same as those used in Experiment 1, except for the learning type, which was self-paced. That is, participants, controlled the amount of time that they spent studying each picture by clicking the left mouse button.

### Results

#### Classification Performance

**Figure 4:**
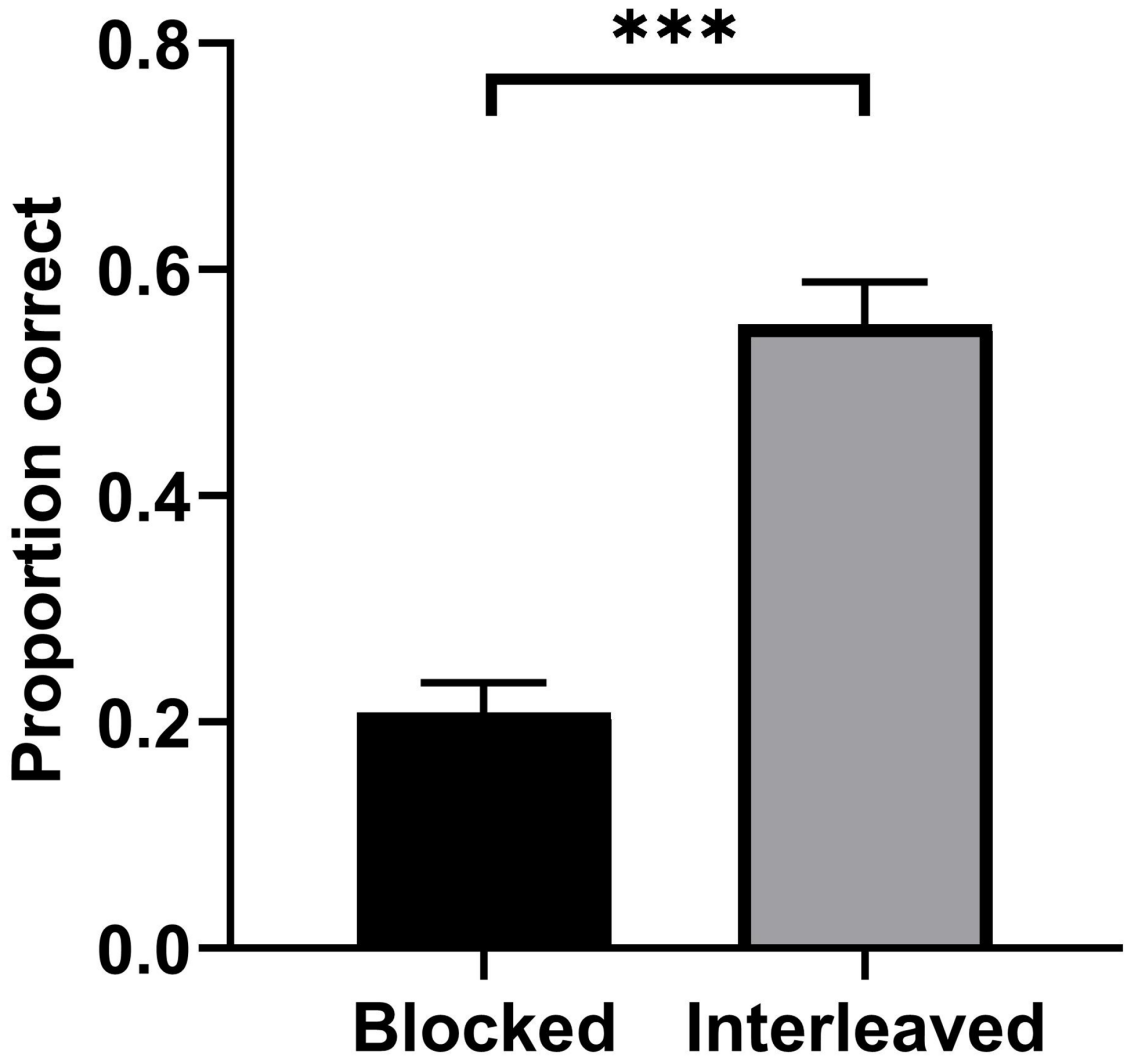
Mean test performance as a function of study condition in Experiment 2. Error bars represent SEM. ^***^p < 0.001.

Classification performance was analyzed using a paired-samples *t*-test. Participants’ performance in interleaved study (*M* = 0.55, *SD* = 0.04) was significantly superior to that observed in blocked study (*M* = 0.21, *SD* = 0.03), *t*(24) = 9.41, *p* < 0.001, *d* = 1.88. Similar to Experiment 1, the results of this experiment indicated that the interleaving effect was present in category learning (Figure 4).

#### Fixation Duration

**Figure 5:**
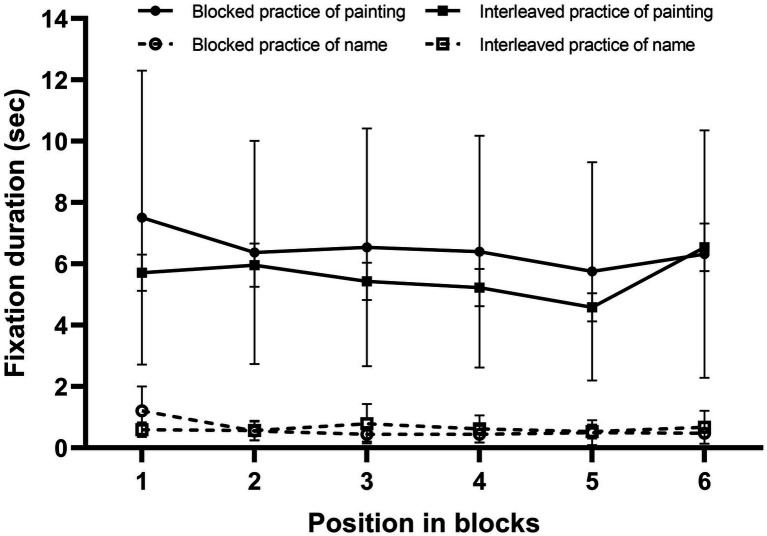
Mean fixation durations for painting and name, as a function of relative position and study condition, in Experiment 2. Error bars represent SE.

Fixation duration was calculated in the manner that was described for Experiment 1. Figure 5 shows the mean fixation durations for the painting and name, as a function of relative position and study condition. An ANOVA was performed, as described for Experiment 1. The main effect of stimulus was significant, *F*(1, 21) = 57.21, *p* < 0.01, *η*^2^ = 0.73. Fixation duration in painting (*M* = 6.03, *SD* = 3.30) was significantly superior to fixation duration in names (*M* = 0.62, *SD* = 0.29). The main effect of study condition was also significant, *F*(1, 21) = 7.69, *p* < 0.05, *η*^2^ = 0.27, reflecting longer fixation duration in blocked study (*M* = 7.08, *SD* = 3.78) relative to interleaved study (*M* = 6.21, *SD* = 3.01). The main effect of exemplar position was significant, *F*(5, 105) = 10.99, *p* < 0.001, *η*^2^ = 0.34. The fixation of Position 1 was significantly longer relative to those observed for Positions 2–6 and the fixation of Position 5 was significantly shorter relative to those observed for Positions 1–4 and 6. The interaction between stimulus and study condition was significant, *F*(1, 21) = 10.35, *p* < 0.01, *η*^2^ = 0.33. Simple effects analysis showed that fixation duration for study condition significantly in painting, *t*(24) = 2.90, *p* < 0.05, *d* = 0.75, in that the fixation duration for blocked study was significantly longer relative to those observed for interleaved study; fixation duration for study condition was not significantly in name, *t*(24) = 0.75, *p* = 0.46, *d* = 0.47. The interaction between stimulus and exemplar position was significant, *F*(5, 105) = 5.73, *p* < 0.01, *η*^2^ = 0.21. Simple effects analysis showed that fixation duration for the six relative positions differed significantly in painting, *F*(5, 120) = 8.30, *p* < 0.05, *η*^2^ = 0.26, Position 1 was significantly longer relative to those observed for Positions 2, 3, 4, and 5; fixation duration for the six relative positions differed significantly in name, *F*(5, 120) = 9.24, *p* < 0.001, *η*^2^ = 0.28, however, Position 1 was significantly shorter relative to those observed for Positions 2, 3, 4, 5, and 6. The interaction between study condition and exemplar position was also significant, *F*(5, 105) = 3.16, *p* = 0.046, *η*^2^ = 0.13. Simple effects analysis showed that fixation duration for the six relative positions differed significantly in blocked study, *F*(5, 120) = 7.98, *p* < 0.05, *η*^2^ = 0.25, in that the fixation duration for Position 1 was significantly longer relative to those observed for Positions 2, 3, 4, 5, and 6; fixation duration for the six relative positions also differed significantly in interleaved study, *F*(5, 120) = 4.13, *p* < 0.001, *η*^2^ = 0.15, and the fixation duration for Position 6 was significantly longer relative to those observed for Positions 1, 2, 3, 4, and 5; however, fixation durations did not differ significantly between Positions 1 and 2. Moreover, the interaction between study condition, stimulus and exemplar position was not significant, *F*(5, 105) = 1.87, *p* = 0.16, *η*^2^ = 0.082.

As shown in Figure 5, a decreasing trend was observed in the blocked study, but not interleaved study. To further determine whether or not attention attenuation in blocked study predicts poorer performance, a correlation analysis between the slopes of the trends across positions 1–5,^1^ and the classification performance was conducted in the blocked study (*r* = −0.318, *p* = 0.12) and interleaved study (*r* = 0.187, *p* = 0.37). Although the result reached no significance, it meant that attention-attenuation may have no or very small effect on interleaved effect. Moreover, the fixation duration in the blocked study was longer, relative to those observed in the interleaved study, which contradicts the attention attenuation hypothesis. In addition, there was a downwards tendency in the blocked study of name, so we also cannot rule out the influence of name’s study on the interleaved effect.

### Discussion

For remedying the shortcomings in Experiment 1, this experiment adopted a self-paced study that subjects can control their learning process. Although there is a decreasing trend was observed in the blocked study, but not interleaved study, the correlation analysis between the slopes of the trends across positions and the classification performance was not significant in the blocked and interleaved study. It is suggested that attention-attenuation may have no or very small effect on interleaved effect. Overall, the results of Experiments 1 and 2, based on eye-tracking data, suggested that attention attenuation may not be the primary mechanism underlying the interleaving effect in category induction.

## Experiment 3

This experiment was conducted to test the benefit of discriminative-contrast between exemplars from different categories to interleaving effect. Participants were asked to view 72 paintings by 12 different artists and learn their styles in three conditions: low-contrast, medium-contrast, and high-contrast. Each condition was randomly assigned four artists’ paintings. Based on the discriminative-contrast hypothesis, studying the differences between different categories is crucial to category induction. Therefore, we evaluated to what extent the degree of discriminative-contrast affects classification performance in category induction tasks, especially in visual material tasks.

### Method

#### Participants and Design

Twenty-nine participants (21 females and eight males) were recruited from Zhe Jiang Normal University. Four participants failed to complete the experiment due to the experimental procedure. Participants received course credit and a gift. The participants’ mean age was 20.88 years (*SD* = 0.43), and all normal or corrected-to-normal visual acuity. A one-way within-subjects design was carried out on the performance of category induction with discriminative-contrast presentation style (low-contrast, medium-contrast, and high-contrast conditions) as within-subject factors.

#### Materials

As in Experiment 1, the materials included 120 landscapes or skyscapes painted by 12 artists. Similarly, there were 24 unrelated questions (e.g., “What is the red dot between Indian women’s eyebrows for?”; “Cinnabar or sandalwood?”) as with Birnbaum et al. (2013). The 12 artists were randomly divided into three groups of four artists each and were represented equally in the low-contrast, medium-contrast, and high-contrast conditions.

#### Procedure

**Figure 6:**
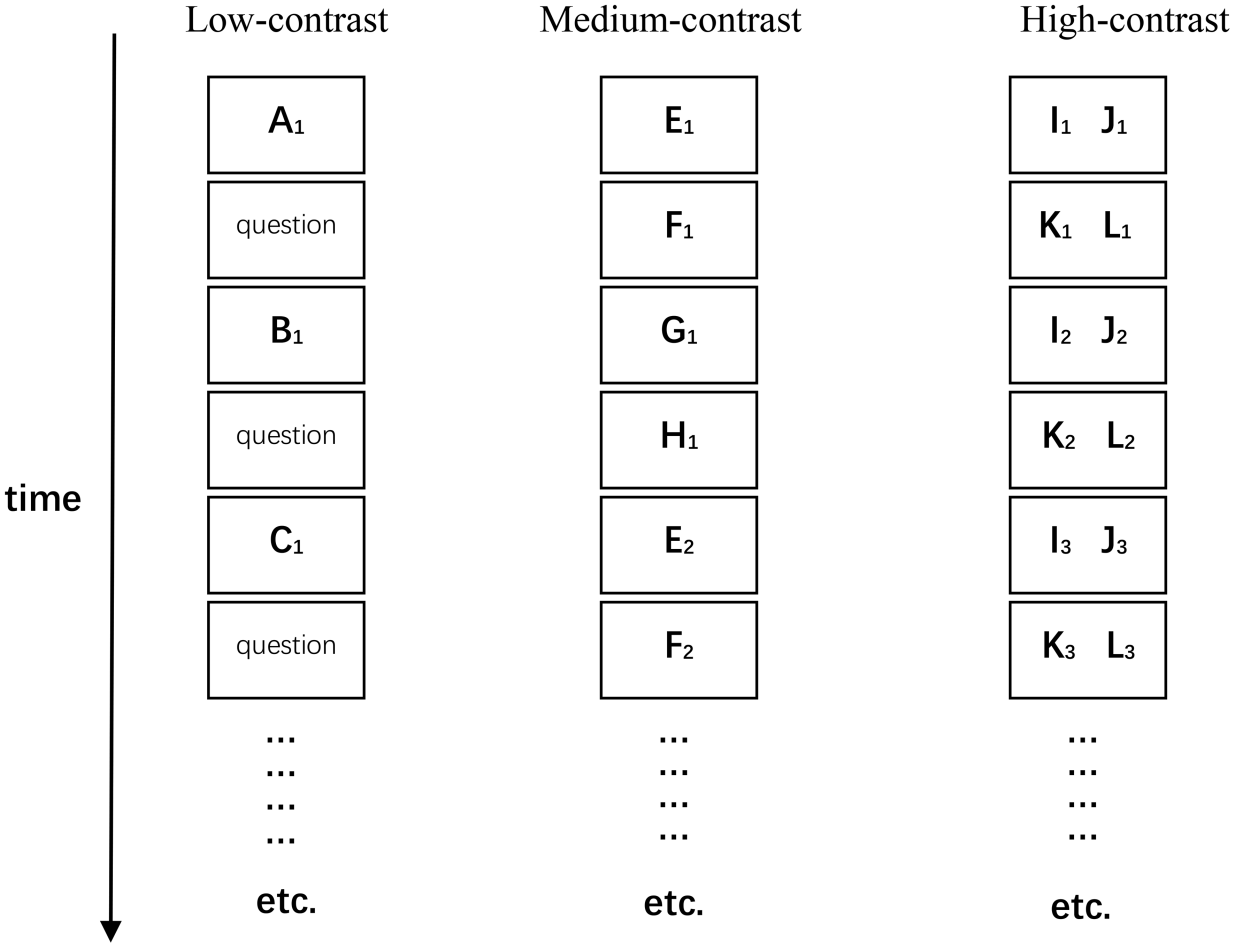
The sequence of paintings during the study phase for the study conditions in Experiment 3. Letters denote a specific artist, and subscript numbers denote a particular painting by that artist.

The experiment was conducted using E-prime 2.0. During the study phase, participants learned the styles of 12 artists by studying six of each of the artists’ paintings (Figure 6). In the low-contrast condition (C_l_), an unrelated question was inserted before each painting was presented, and participants studied the artist’s style as well as answered the unrelated questions (see Experiment 1 in Birnbaum et al., 2013). The medium-contrast condition (C_m_) was conducted in the same way as the low-contrast condition, except that no unrelated question was inserted. In the high-contrast condition (C_h_), the paintings were presented two at a time on the computer screen (see Experiment 1 in Kang and Pashler, 2012). Each painting was shown for 3 s in low-contrast and medium-contrast conditions, and for 6 s in high-contrast conditions. The artist’s name was displayed below the paintings. The order of the blocks was C_l_-C_m_-C_h_-C_m_-C_h_-C_l_-C_h_-C_l_-C_m_-C_m_-C_l_-C_h_-C_l_-C_h_-C_m_-C_h_-C_m_-C_l_. The test phase of the experiment was the same as for Experiments 1 and 2.

### Results

**Figure 7:**
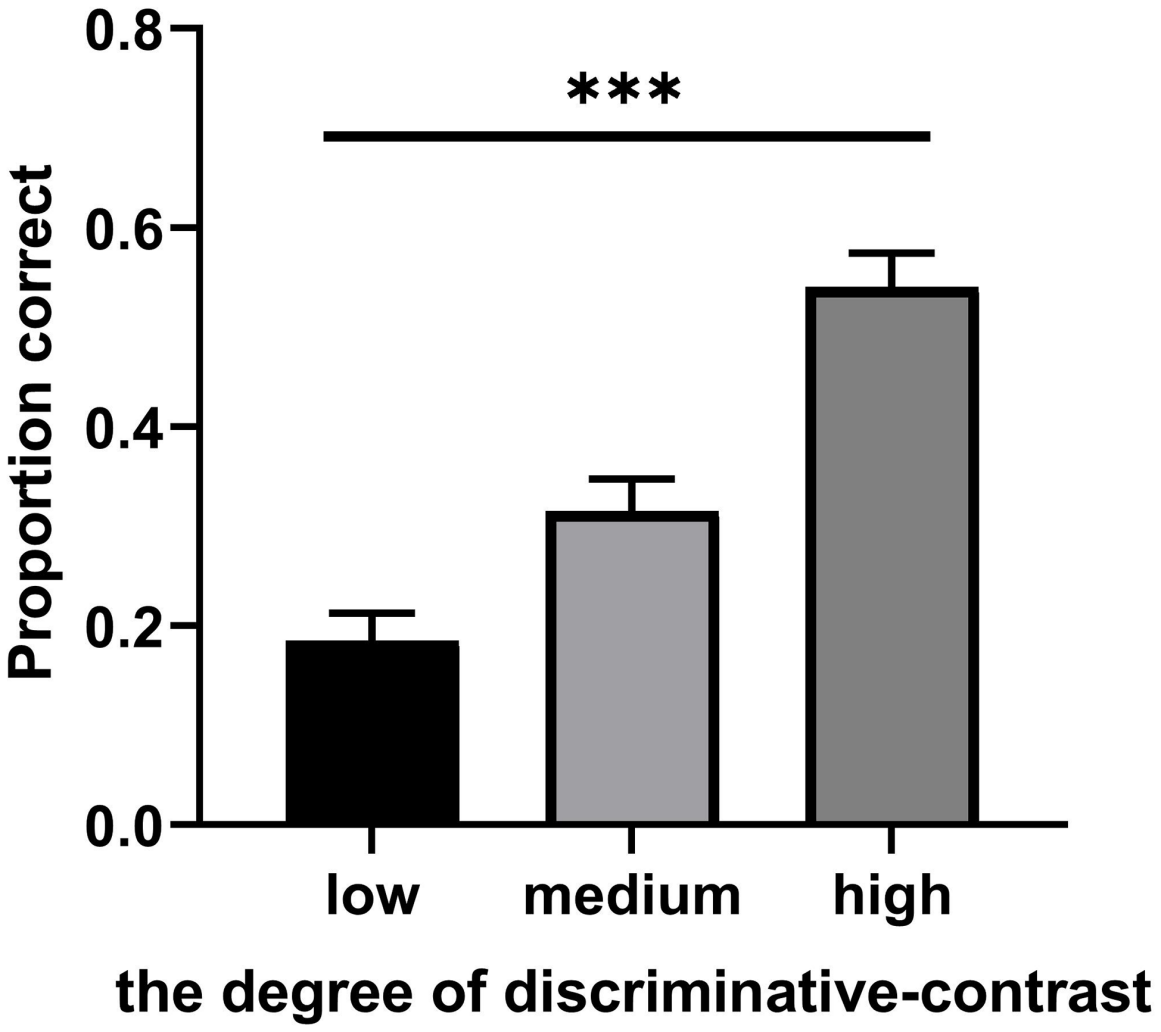
Mean test performance as a function of the degree of discriminative-contrast in Experiment 3. Error bars represent SEM. ^***^p < 0.001.

Figure 7 shows the mean test performance as a function of the degree of discriminative-contrast. A one-way ANOVA on performance on category induction indicated significant differences between the low-contrast condition, the medium-contrast condition, and the high-contrast condition, *F*(2, 48) = 52.04, *p* < 0.001, *η*_p_^2^ = 0.68. The category induction performance in the high-contrast condition (*M* = 0.54, *SD* = 0.17, 95% CI [0.47, 0.61]) was significantly higher than that in the medium-contrast condition (*M* = 0.32, *SD* = 0.16, 95% CI [0.25, 0.38]). Classification performance in the medium-contrast condition was significantly higher than that in the low-contrast condition (*M* = 0.19, *SD* = 0.14, 95% CI [0.13, 0.24]). Therefore, the results of the current investigation support the discriminative-contrast hypothesis.

### Discussion

This experiment tests the discriminative-contrast hypothesis by manipulating the degree of discriminative-contrast. The results showed that the degree of discriminative-contrast had significant effects on classification performance, that is, classification performance in the high-contrast condition was significantly higher than that in the medium-contrast and low-contrast condition, which support the discriminative-contrast hypothesis. The results obtained were consistent with those of other studies (Kang and Pashler, 2012). Further, an unrelated question was inserted before each painting was presented in the low-contrast condition, in fact, this will increase the sample learning interval. Previous studies have found that the appropriate learning interval can promote learning performance (Cepeda et al., 2006). Even so, the classification performance in the low-contrast condition was significantly lower than that in the medium-contrast and high-contrast conditions. Thus, the results of the current experiment support the discriminative-contrast hypothesis.

## General Discussion

What is the mechanism underlying the interleaving effect in category induction? The current study evaluated the attention attenuation hypothesis and the discriminative-contrast hypothesis from the perspective of “why blocked is worse?” and “why interleaved is better?”, respectively. The eye-tracking results indicated that fixation durations for the six relative positions did not differ significantly for blocked or interleaved practices and the correlation between the slopes of the trends across positions and the classification performance was not significant in blocked and interleaved study, but the classification performance in the high-contrast condition was significantly higher than in the medium-contrast and low-contrast conditions. Three experiments provide evidence to support the discriminative-contrast hypothesis rather than attention attenuation hypothesis in terms of category induction, especially for visual material tasks.

It may be reasonable to conclude that the attention attenuation hypothesis might not be fully applicable in explaining the interleaving effect in category induction, at least in complex visual stimuli such as paintings. This is somewhat inconsistent with findings such as those of Wahlheim et al. (2011), who regarded memory performance for items that had been studied as an indicator of attention paid to which position in the study sequence the item had appeared. Wahlheim et al. (2011) assumed that better memory performance indicated higher levels of attention allocation. The results suggested that memory performance for samples of each position within a block decreases as a function of the relative position under the condition of blocked practice. Conversely, no such decrease will arise for interleaved practice blocks. An interpretation of these results is that learners did not attend to the later presentations of items in the blocked sequence to the same extent as the earlier ones, and, therefore, do not recall them as readily. Although this was a sophisticated experiment, caution should be exercised in adopting this hypothesis, as memory performance is influenced by several other factors, including the sequence in which the stimulus is presented, and the primacy and recency effects. Therefore, differences in category induction performance might not have reflected differences in the allocation of attentional resources. The indirect nature of this indicator may be the reason for the differences from the current research results.

Fixation duration is believed to be a more direct and accurate measure of attentional resource allocation than category memory performance (Kruschke et al., 2005; Rehder and Hoffman, 2005b; Zaki and Salmi, 2019). Thus, using this indicator to evaluate the attention attenuation hypothesis may be more reliable. Furthermore, the attention-attenuation hypothesis was originally used to explain the interleaving effect in repetition-based learning, such as that involving words and word pairs, and was validated in studies involving the repeated presentation of the same stimulus (Cepeda et al., 2006; Ariel et al., 2014; Mulligan and Peterson, 2014). However, category induction differs from repetition-based learning. In category induction, exemplars belonging to the same category possess distinctive characteristics. Thus, in blocked practice, exemplars from the same category have the same features, but their specific content differs. Therefore, each exemplar carries some degree of novelty for the participants. In the current study, each painting was presented for only 3 s in the fixed-paced learning in Experiment 1, and memory coding could have been insufficient for some stimuli. Therefore, participants might not have had time to fully focus on the stimulus and the opportunity to reduce their attention. Experiment 2 allows participants sufficient time to encode the paintings fully by using the self-paced study as opposed to fixed-paced learning. Although a decreasing trend was observed in the blocked study, but not interleaved study. The correlation between the slopes of the trends across positions and the classification performance was not significant in blocked and interleaved studies. Thus, it is suggested that attention attenuation may not be the primary mechanism underlying the interleaving effect in category induction.

The current finding that the degree of discriminative-contrast affects classification performance strongly supports the discriminative-contrast hypothesis, namely, an interleaved practice should generate a higher performance of category learning in that exemplars of different categories within the same domain (e.g., artists’ landscapes) are presented in close proximity, which is beneficial for participants to observe the similarities and differences across the categories (Kornell and Bjork, 2008; Kornell et al., 2010; Kang and Pashler, 2012; Zulkiply and Burt, 2013; Carvalho and Goldstone, 2014). These findings are consistent with those of Kang and Pashler (2012) who replicated Kornell and Bjork (2008) and Kornell et al. (2010) results and suggested that the benefits of interleaved practice are tied to the temporal contiguity among items of different concepts, or that require different responses, as opposed to the increased temporal lag between exemplars’ presence in the same category. However, Foster et al. (2019) evaluated the discriminative-contrast hypothesis and the distributed-practice hypothesis by examing the participants’ performance on the solving of mathematics problems, that is, calculating the volume of three-dimensional geometric shapes. They found that the interleaving effects on the solution of geometry problems are largely driven by distributed practice rather than the discriminative-contrast hypothesis. A comprehensive meta-analysis of studies on the effects of interleaved learning found significant differences depending on the types of learning materials (Brunmair and Richter, 2019). Interleaving had a positive effect on category induction for all types of visual stimuli, with the highest mean effect being for paintings and naturalistic photographs. In nonvisual stimuli, a positive interleaving effect was found only for mathematical tasks, and no significant effect was found for expository texts (Brunmair and Richter, 2019). This seems to suggest that the cognitive processes underlying the interleaved practice of visual and text materials may differ. Visual materials (e.g., Kang and Pashler, 2012) are more affected by discriminative-contrast, while expository texts materials (e.g., Foster et al., 2019) are more affected by distributed-practice. Further research is required to clarify these differences.

In response to equivocal findings, Carvalho and Goldstone (2019) proposed the Sequential Attention Theory (SAT), which attempts to explain this inconsistency. In light of this theory, interleaved and blocked practice arrangements highlight different aspects of the category’s learning material. If there is a greater similarity between categories, there is a more positive influence on the interleaving effect. This is due to difficulties in identifying category features. The interleaved practice could have similar samples from different categories being successively presented, which allows individuals to identify the diagnostic features that make categories different from each other more easily. In other words, the differences between categories are highlighted as opposed to the similarities. On the other hand, if there is a lower level similarity within-category, then there is a negative influence on the interleaving effect because it is more difficult to identify exemplar’s shared features for the same category. In this case, interleaved practice is not conducive to category learning since samples from the same categories are distributed, which may prevent participants from learning the features inherent to similar categories (Foster et al., 2019). Therefore, the differences in category structure may play an important role in the inconsistencies seen in research findings (Chin-Parker and Ross, 2002).

The materials used in the current study, and that of Kang and Pashler (2012), were artists’ paintings, which exhibit relatively high similarities between categories. Discriminative-contrast is more conducive to participants identifying the diagnostic features that separate categories as different from each other. Conversely, in Foster et al. (2019), the materials used were the formulae for calculating the volume of four three-dimensional geometric shapes (i.e., wedges, spheroids, spherical cones, and half cones), which represent lower levels of similarity between categories since each shape is distinct. In these cases, discriminative-contrast between the shapes may be a relatively trivial task, since participants can quickly and easily distinguish between the four shape types. Interestingly, categorical items are temporally spaced in interleaved practice, because of distributed presentation. This means that interleaving effects might be partially due to a spacing effect (Kornell and Bjork, 2008; Wahlheim et al., 2011), which benefits participants in establishing associations between each shape and its formula (Janiszewski et al., 2003; Cepeda et al., 2006, 2009; Benjamin and Tullis, 2010; Carpenter et al., 2012; Küpper-Tetzel, 2014). Thus, the interleaving effect in Foster et al. (2019) may have been facilitated by distributed practice rather than only discriminative-contrast. In general, the observed inconsistencies in findings regarding discriminative-contrast may partly be due to the material properties and the characteristics of the category structures.

The current study extended the findings of previous research examining the mechanisms underlying the interleaving effect in category induction, using two experiments. First, fixation duration was used as a direct attention index to test the attention attenuation hypothesis in a category induction task. Second, the attention attenuation hypothesis was examined with the intention of obtaining reliable evidence given the current equivocal findings in this field. The results indicated that the attention attenuation hypothesis was not fully applicable in explaining the interleaving effect in category induction, but an argument was made for discriminative-contrast to have produced the interleaving effect in category induction. However, we cannot dismiss the theoretical value of the attention attenuation hypothesis, as it provides a powerful explanation for the interleaving effect, particularly in repetition-based learning (Ariel et al., 2014).

Although numerous implications exist, there are also some study limitations. Firstly, the order of the blocks was B-I-I-B-B-I-I-B-B-I-I-B alone. Thus, the blocked (B) study’s block was always learned first. This may be a potential confound to the allocation of attention. Future research should add alternative orders for more comprehensive study (e.g., I-B-B-I-I-B-B-I-I-B-B-I), and counterbalance these effects. Future research should also consider the effect of ordering within categories as influential in the allocation of attention. Secondly, the manipulation of the degree of discriminative-contrast invites alternative hypotheses. For instance, an unrelated question was inserted before each painting was presented in low-contrast condition will no doubt make participants more distracted. The paintings were presented two at a time on the computer screen in high-contrast conditions leads to deeper processing because subjects may not spend an average amount of time on each painting. Finally, the differences in category structure may play an important role in category induction; however, we did not assess the characteristics of the experimental materials (e.g., landscapes or skyscapes painted by 12 artists).

## Data Availability Statement

The raw data supporting the conclusions of this article will be made available by the authors, without undue reservation.

## Ethics Statement

The studies involving human participants were reviewed and approved by The Zhejiang Normal University Review Board. The patients/participants provided their written informed consent to participate in this study.

## Author Contributions

YG designed this study, collected the data, analyzed the data, and wrote the manuscript. FL and WL proposed the research idea and demonstrate the feasibility of the method. XL participates in language polishing to ensure manuscript quality and joined the data analysis. All authors contributed to the article and approved the submitted version.

## Funding

This research was supported by the Ministry of Education in China (MOE) Project of Humanities and Social Sciences (project no. 19YJA190003) and the Open Research Fund of College of Teacher Education, Zhejiang Normal University (no. JYKF20002) to XL.

## Conflict of Interest

The authors declare that the research was conducted in the absence of any commercial or financial relationships that could be construed as a potential conflict of interest.

## Publisher’s Note

All claims expressed in this article are solely those of the authors and do not necessarily represent those of their affiliated organizations, or those of the publisher, the editors and the reviewers. Any product that may be evaluated in this article, or claim that may be made by its manufacturer, is not guaranteed or endorsed by the publisher.

## Footnotes

^1^The *R*^2^ of linear regression of positions 1–6 was so low that researchers selected positions 1–5.

## References

[ref1] Ariel R. Dunlosky J. Toppino T. C. (2014). Contribution of degraded perception and insufficient encoding to decisions to mass or space study. Exp. Psychol. 61, 110–117. doi: 10.1027/1618-3169/a000230, PMID: 23988872

[ref2] Ashby F. G. O'Brien J. B. (2005). Category learning and multiple memory systems. Trends Cogn. Sci. 9, 83–89. doi: 10.1016/j.tics.2004.12.003, PMID: 15668101

[ref3] Benjamin A. S. Tullis J. (2010). What makes distributed practice effective? Cogn. Psychol. 61, 228–247. doi: 10.1016/j.cogpsych.2010.05.004, PMID: 20580350 PMC2930147

[ref4] Birnbaum M. S. Kornell N. Bjork E. L. Bjork R. A. (2013). Why interleaving enhances inductive learning: The roles of discrimination and retrieval. Mem. Cogn. 41, 392–402. doi: 10.3758/s13421-012-0272-7, PMID: 23138567

[ref5] Brunmair M. Richter T. (2019). Similarity matters: a meta-analysis of interleaved learning and its moderators. Psychol. Bull. 145, 1029–1052. doi: 10.1037/bul0000209, PMID: 31556629

[ref6] Carpenter S. K. Cepeda N. J. Rohrer D. Kang S. H. K. Pashler H. (2012). Using spacing to enhance diverse forms of learning: review of recent research and implications for instruction. Educ. Psychol. Rev. 24, 369–378. doi: 10.1007/s10648-012-9205-z

[ref7] Carvalho P. F. Goldstone R. L. (2014). Putting category learning in order: category structure and temporal arrangement affect the benefit of interleaved over blocked study. Mem. Cogn. 42, 481–495. doi: 10.3758/s13421-013-0371-0, PMID: 24092426

[ref8] Carvalho P. F. Goldstone R. L. (2017). The sequence of study changes what information is attended to, encoded, and remembered during category learning. J. Exp. Psychol. Learn. Mem. Cogn. 43, 1699–1719. doi: 10.1037/xlm0000406, PMID: 28333507

[ref9] Carvalho P. F. Goldstone R. L. (2019). “When does interleaving practice improve learning?” in The Cambridge Handbook of Cognition and Education. eds. Dunlosky J. Rawson K. A. (Cambridge: Cambridge University Press), 411–436.

[ref10] Cepeda N. J. Coburn N. Rohrer D. Wixted J. T. Mozer M. C. Pashler H. (2009). Optimizing distributed practice: theoretical analysis and practical implications. Exp. Psychol. 56, 236–246. doi: 10.1027/1618-3169.56.4.236, PMID: 19439395

[ref11] Cepeda N. J. Pashler H. Vul E. Wixted J. T. Rohrer D. (2006). Distributed practice in verbal recall tasks: a review and quantitative synthesis. Psychol. Bull. 132, 354–380. doi: 10.1037/0033-2909.132.3.354, PMID: 16719566

[ref12] Chin-Parker S. Ross B. H. (2002). The effect of category learning on sensitivity to within-category correlations. Mem. Cogn. 30, 353–362. doi: 10.3758/BF03194936, PMID: 12061756

[ref13] Dienes Z. (2014). Using Bayes to get the most out of non-significant results. Front. Psychol. 5:781. doi: 10.3389/fpsyg.2014.00781, PMID: 25120503 PMC4114196

[ref14] Dunlosky J. Rawson K. A. Marsh E. J. Nathan M. J. Willingham D. T. (2013). Improving students’ learning with effective learning techniques: promising directions from cognitive and educational psychology. Psychol. Sci. Public Interest 14, 4–58. doi: 10.1177/1529100612453266, PMID: 26173288

[ref15] Eglington L. G. Kang S. H. K. (2017). Interleaved presentation benefits science category learning. J. Appl. Res. Mem. Cogn. 6, 475–485. doi: 10.1016/j.jarmac.2017.07.005, PMID: 31900048

[ref16] Faul F. Erdfelder E. Buchner A. Lang A.-G. (2009). Statistical power analyses using G*power 3.1: tests for correlation and regression analyses. Behav. Res. Methods 41, 1149–1160. doi: 10.3758/BRM.41.4.1149, PMID: 19897823

[ref17] Faul F. Erdfelder E. Lang A.-G. Buchner A. (2007). GPower 3: a flexible statistical power analysis program for the social, behavioral, and biomedical sciences. Behav. Res. Methods 39, 175–191. doi: 10.3758/BF03193146, PMID: 17695343

[ref18] Foster N. L. Mueller M. L. Was C. Rawson K. A. Dunlosky J. (2019). Why does interleaving improve math learning? The contributions of discriminative contrast and distributed practice. Mem. Cogn. 47, 1088–1101. doi: 10.3758/s13421-019-00918-4, PMID: 30877483

[ref19] Guzman-Munoz F. J. (2016). The advantage of mixing examples in inductive learning: a comparison of three hypotheses. Educ. Psychol. 37, 421–437. doi: 10.1080/01443410.2015.1127331

[ref20] Hintzman D. L. (1974). “Theoretical implications of the spacing effect,” in Theories in Cognitive Psychology: The Loyola Symposium. ed. Solso R. L. (Oxford, England: Lawrence Erlbaum).

[ref21] Hu Chuan-Peng K. X.-Z. Eric-Jan W. Alexander L. Y. Kaiping P. (2018). The Bayes factor and its implementation in JASP: a practical primer. Adv. Psychol. Sci. 26, 951–965. doi: 10.3724/sp.J.1042.2018.00951

[ref22] Janiszewski C. Noel H. Sawyer A. G. (2003). A meta-analysis of the spacing effect in verbal learning: implications for research on advertising repetition and consumer memory. J. Consum. Res. 30, 138–149. doi: 10.1086/374692

[ref23] Jarosz A. F. Wiley J. (2014). What are the odds? A practical guide to computing and reporting Bayes factors. J. Probl. Solving 7, 2–9. doi: 10.7771/1932-6246.1167

[ref25] Kang S. H. K. Pashler H. (2012). Learning painting styles: spacing is advantageous when it promotes discriminative contrast. Appl. Cogn. Psychol. 26, 97–103. doi: 10.1002/acp.1801

[ref26] Kornell N. Bjork R. A. (2008). Learning concepts and categories: is spacing the “enemy of induction”? Psychol. Sci. 19, 585–592. doi: 10.1111/j.1467-9280.2008.02127.x, PMID: 18578849

[ref27] Kornell N. Castel A. D. Eich T. S. Bjork R. A. (2010). Spacing as the friend of both memory and induction in young and older adults. Psychol. Aging 25, 498–503. doi: 10.1037/a0017807, PMID: 20545435

[ref28] Kruschke J. K. (2005). “Category induction,” in The Handbook of Cognition. eds. Lamberts K. Goldstone R. L. (London: Sage), 183–201.

[ref29] Kruschke J. K. Kappenman E. S. Hetrick W. P. (2005). Eye gaze and individual differences consistent with learned attention in associative blocking and highlighting. J. Exp. Psychol. Learn. Mem. Cogn. 31, 830–845. doi: 10.1037/0278-7393.31.5.830, PMID: 16248737

[ref30] Küpper-Tetzel C. E. (2014). Understanding the distributed practice effect. Z. Psychol. 222, 71–81. doi: 10.1027/2151-2604/a000168

[ref31] Markman A. B. Ross B. H. (2003). Category use and category learning. Psychol. Bull. 129, 592–613. doi: 10.1037/0033-2909.129.4.592, PMID: 12848222

[ref32] Marsman M. Wagenmakers E.-J. (2017). Bayesian benefits with JASP. Eur. J. Dev. Psychol. 14, 545–555. doi: 10.1080/17405629.2016.1259614

[ref33] Mathy F. Feldman J. (2016). The influence of presentation order on category transfer. Exp. Psychol. 63, 59–69. doi: 10.1027/1618-3169/a000312, PMID: 27025535

[ref34] Metcalfe J. Xu J. (2016). People mind wander more during massed than spaced inductive learning. J. Exp. Psychol. Learn. Mem. Cogn. 42, 978–984. doi: 10.1037/xlm0000216, PMID: 26618908

[ref35] Mulligan N. W. Peterson D. J. (2014). The spacing effect and metacognitive control. J. Exp. Psychol. Learn. Mem. Cogn. 40, 306–311. doi: 10.1037/a0033866, PMID: 23895449

[ref36] Rehder B. Hoffman A. B. (2005a). Eyetracking and selective attention in category learning. Cogn. Psychol. 51, 1–41. doi: 10.1016/j.cogpsych.2004.11.001, PMID: 16039934

[ref37] Rehder B. Hoffman A. B. (2005b). Thirty-something categorization results explained: selective attention, eyetracking, and models of category learning. J. Exp. Psychol. Learn. Mem. Cogn. 31, 811–829. doi: 10.1037/0278-7393.31.5.811, PMID: 16248736

[ref38] Rohrer D. (2012). Interleaving helps students distinguish among similar concepts. Educ. Psychol. Rev. 24, 355–367. doi: 10.1007/s10648-012-9201-3

[ref39] Rohrer D. Dedrick R. F. Stershic S. (2015). Interleaved practice improves mathematics learning. J. Educ. Psychol. 107, 900–908. doi: 10.1037/edu0000001, PMID: 24578089

[ref40] Sana F. Yan V. X. Kim J. A. (2017). Study sequence matters for the inductive learning of cognitive concepts. J. Educ. Psychol. 109, 84–98. doi: 10.1037/edu0000119

[ref41] Taylor K. Rohrer D. (2010). The effects of interleaved practice. Appl. Cogn. Psychol. 24, 837–848. doi: 10.1002/acp.1598, PMID: 34375844

[ref42] Verkoeijen P. P. Bouwmeester S. (2014). Is spacing really the "friend of induction"? Front. Psychol. 5:259. doi: 10.3389/fpsyg.2014.00259, PMID: 24744742 PMC3978334

[ref43] Vlach H. A. Sandhofer C. M. Kornell N. (2008). The spacing effect in children's memory and category induction. Cognition 109, 163–167. doi: 10.1016/j.cognition.2008.07.013, PMID: 18835602

[ref44] Wagenmakers E.-J. Lodewyckx T. Kuriyal H. Grasman R. (2010). Bayesian hypothesis testing for psychologists: a tutorial on the Savage–Dickey method. Cogn. Psychol. 60, 158–189. doi: 10.1016/j.cogpsych.2009.12.001, PMID: 20064637

[ref45] Wahlheim C. N. Dunlosky J. Jacoby L. L. (2011). Spacing enhances the learning of natural concepts: an investigation of mechanisms, metacognition, and aging. Mem. Cogn. 39, 750–763. doi: 10.3758/s13421-010-0063-y, PMID: 21264639 PMC3085105

[ref46] Yamauchi T. Markman A. B. (2000). Inference using categories. J. Exp. Psychol. Learn. Mem. Cogn. 26, 776–795. doi: 10.1037/0278-7393.26.3.776, PMID: 10855431

[ref47] Zaki S. R. Salmi I. L. (2019). Sequence as context in category learning: an eyetracking study. J. Exp. Psychol. Learn. Mem. Cogn. 45, 1942–1954. doi: 10.1037/xlm0000693, PMID: 30816766

[ref48] Zechmeister E. B. Shaughnessy J. J. (1980). When you know that you know and when you think that you know but you don't. Bull. Psychon. Soc. 15, 41–44. doi: 10.3758/BF03329756, PMID: 34653066

[ref49] Zulkiply N. Burt J. S. (2013). The exemplar interleaving effect in inductive learning: moderation by the difficulty of category discriminations. Mem. Cogn. 41, 16–27. doi: 10.3758/s13421-012-0238-9, PMID: 22886736

[ref50] Zulkiply N. McLean J. Burt J. S. Bath D. (2012). Spacing and induction: application to exemplars presented as auditory and visual text. Learn. Instr. 22, 215–221. doi: 10.1016/j.learninstruc.2011.11.002

